# Sources and Levels of Ambient Ocean Sound near the Antarctic Peninsula

**DOI:** 10.1371/journal.pone.0123425

**Published:** 2015-04-14

**Authors:** Robert P. Dziak, DelWayne R. Bohnenstiehl, Kathleen M. Stafford, Haruyoshi Matsumoto, Minkyu Park, Won Sang Lee, Matt J. Fowler, Tai-Kwan Lau, Joseph H. Haxel, David K. Mellinger

**Affiliations:** 1 Oregon State University/Cooperative Institute for Marine Resources Studies and NOAA/Pacific Marine Environmental Laboratory, Newport, Oregon, 97365–5258, United States of America; 2 Department of Marine, Earth and Atmospheric Sciences, North Carolina State University, Raleigh, North Carolina, 27695–8208, United States of America; 3 Applied Physics Laboratory, University of Washington, Seattle, Washington, United States of America; 4 Polar Environmental Research Division, Korea Polar Research Institute, Incheon 406–840, Republic of Korea; Virginia Commonwealth Univ, UNITED STATES

## Abstract

Arrays of hydrophones were deployed within the Bransfield Strait and Scotia Sea (Antarctic Peninsula region) from 2005 to 2009 to record ambient ocean sound at frequencies of up to 125 and 500 Hz. Icequakes, which are broadband, short duration signals derived from fracturing of large free-floating icebergs, are a prominent feature of the ocean soundscape. Icequake activity peaks during austral summer and is minimum during winter, likely following freeze-thaw cycles. Iceberg grounding and rapid disintegration also releases significant acoustic energy, equivalent to large-scale geophysical events. Overall ambient sound levels can be as much as ~10–20 dB higher in the open, deep ocean of the Scotia Sea compared to the relatively shallow Bransfield Strait. Noise levels become lowest during the austral winter, as sea-ice cover suppresses wind and wave noise. Ambient noise levels are highest during austral spring and summer, as surface noise, ice cracking and biological activity intensifies. Vocalizations of blue (*Balaenoptera musculus*) and fin (*B*. *physalus*) whales also dominate the long-term spectra records in the 15–28 and 89 Hz bands. Blue whale call energy is a maximum during austral summer-fall in the Drake Passage and Bransfield Strait when ambient noise levels are a maximum and sea-ice cover is a minimum. Fin whale vocalizations were also most common during austral summer-early fall months in both the Bransfield Strait and Scotia Sea. The hydrophone data overall do not show sustained anthropogenic sources (ships and airguns), likely due to low coastal traffic and the typically rough weather and sea conditions of the Southern Ocean.

## Introduction

The climate of the Antarctic Peninsula is changing in the Southern Hemisphere, with a few degrees Celsius rise in both atmospheric and surface ocean temperatures over the last few decades [1–3]. Associated with this ongoing warming is a cycle of ice sheet and iceberg breakup and grounding that is accompanied by the release of acoustic energy into the Southern Ocean. Although much attention has been given to the increasing anthropogenic contributions to ocean noise elsewhere in the world, which may be as much as 12 dB relative to historical (1960s) levels [4], the sounds created by ice breakup in the Southern Ocean may represent an underappreciated, yet significant, natural contribution to the ocean noise budget.

To further study these ice-related and other sources of natural sound, we deployed arrays of hydrophone moorings in the Bransfield Strait, a protected sea along the western Antarctic Peninsula, and the Scotia Sea, an area of the South Atlantic northeast of the Antarctic Peninsula. A variety of cryogenic sounds were recorded, associated with iceberg grounding and breakup. Icequakes are impulsive broadband signals with durations between 10 and 80 sec and dominant energy over the ~10–500 Hz band. Icequakes may be caused by thermal stresses, as well as physical deformation of the ice from wind, currents, and waves, which can cause fracturing and colliding of large ice blocks and bergs, producing sound [5,6]. These external forcing factors result in shear failure of the ice crystal lattice, generating pressure waves that emanate into the water column. Studies in the Arctic have shown there is also seasonal variation to ice fracture events, where wind speeds and the amount of ice coverage versus open water dictate long-term ambient noise levels [7]. During this experiment, we recorded the acoustic signals produced by iceberg A53a (initially ~1100 km^2^ in area), as it drifted from its origin in the Weddell Sea, grounded within the Bransfield Strait, and broke apart in the Scotia Sea.

Other major components of the ambient sound field near the Antarctic Peninsula are the low-frequency vocalizations of large baleen whales, which dominate the 15–28 and 89 Hz frequency bands [8–10] and the waterborne acoustic phases of seafloor earthquakes, which primarily exhibit energy over the 1–50 Hz band (e.g., [11]). The goal of this paper is to present the various sources of ambient sound in these regions and estimate month to year-long variability in acoustic energy levels to identify what processes characterize and influence the soundscape in this part of the Southern Ocean.

## Materials and Methods

**Fig 1:**
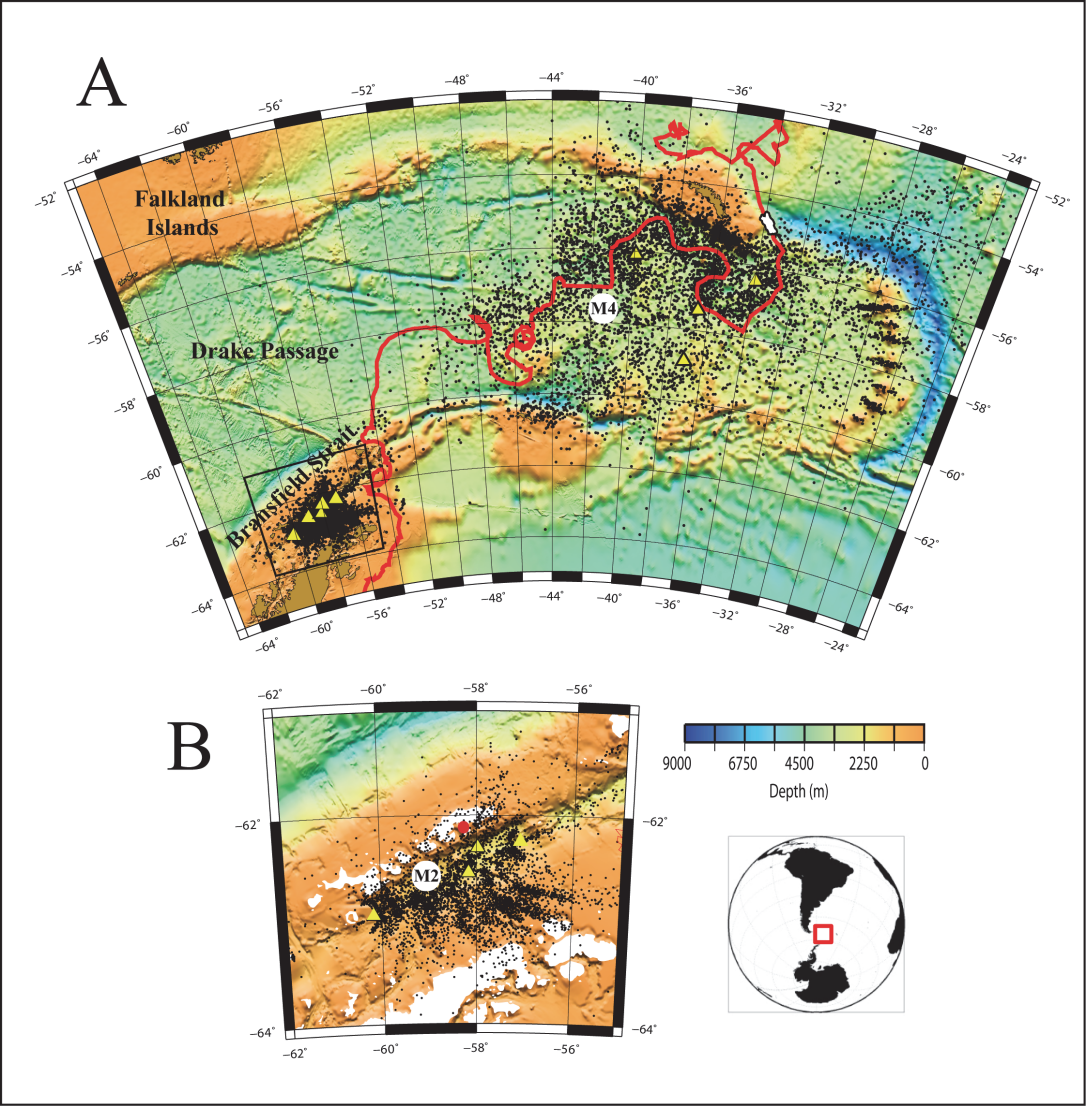
Geographic location of the Antarctic Peninsula (AP), Bransfield Strait (BS), Drake Passage (DP), and Scotia Sea. Yellow triangles show 2005–2007 and 2008–2009 hydrophone mooring deployments within Bransfield Strait (elongated basin northwest of AP) and Drake Passage (B), as well as the 2007–2008 deployments within Scotia Sea (A). Circles M2 and M4 show locations of hydrophone moorings used to make spectrograms in Fig 6. Black dots show regional icequakes located using both hydrophone arrays. All events shown were located using 4–5 hydrophones. Red line shows 8-month track of iceberg A53a from Weddell to Scotia seas derived from NASA’s Quick-Scatterometer (QuicScat) satellite (BYU Scatterometer Climate Record Pathfinder; www.scp.byu.edu). White polygon shows approximate shape and size of A53a when near South Georgia Island on 12 February 2008 (Fig 5). Positions “1” and “2” in red show iceberg locations where harmonic tremor (IHT) was produced. Bathymetry is from Sandwell and Smith [49] satellite altimetry.

In order to monitor the levels and determine the sources of acoustic energy near the Antarctic Peninsula, two arrays of hydrophone instrument moorings were deployed in the Bransfield Strait and Scotia Sea (Fig 1A and 1B). The Bransfield Strait arrays consisted of five and six hydrophone moorings deployed December 2005–2007 and November 2008–December 2009, respectively [11,12]. One hydrophone mooring was deployed in the Drake Passage, north of the Bransfield Strait, during December 2005 to December 2006. A total of five hydrophones were deployed in the Scotia Sea from December 2007 to December 2008.

**Fig 2:**
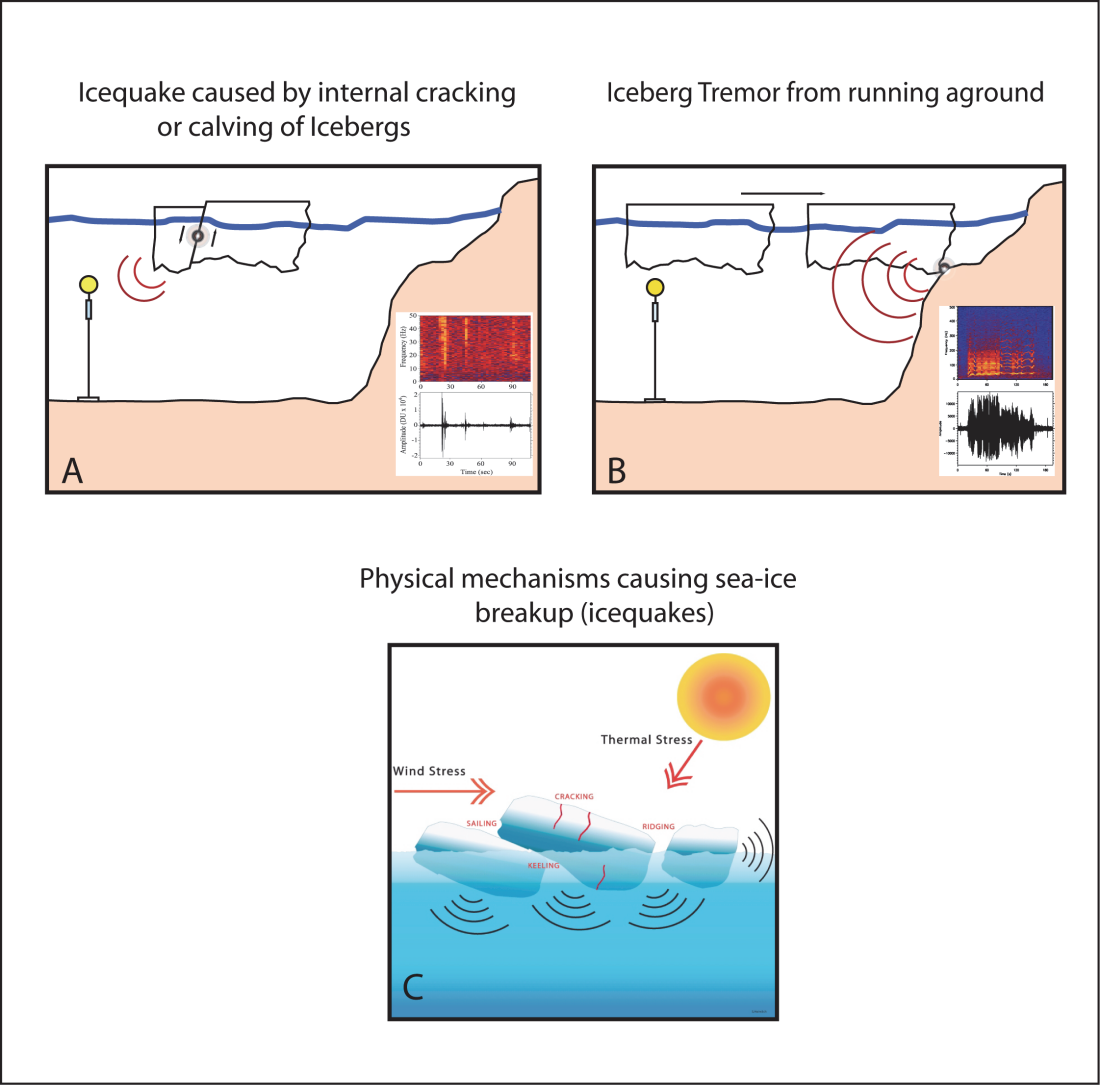
Diagrams showing how iceberg sounds couple into the ocean and are recorded at the hydrophone. Also shown are time series and spectrogram of hydrophone data examples of an (A) icequake and (B) iceberg harmonic tremor. (C) The various physical causes of sound generation from sea ice (after [5,6]). Thermal stress (either due to thawing or freezing) and wind can cause fracturing and colliding of ice blocks, producing sound. Icequakes are distinguished from earthquakes by their impulsive, short-duration coda (~10 s) and higher-frequency content (>10–50 Hz). Iceberg tremor can be caused by either grounding of the iceberg on the shelf or contact with another iceberg, creating acoustics waves via stick-slip behavior and vibration [25]. A float (yellow circle) keeps the hydrophone (cylinder) and mooring line aloft in the water column; line is moored to the seafloor via an acoustic release and anchor.

The autonomous hydrophone instrument package consists of a single ceramic hydrophone, a filter/amplifier stage, an accurate clock, a low-power processor, and a battery package. The instrument is capable of recording at 16-bit data resolution between 250 and 1000 Hz for periods of 1 to 2.5 years, depending on sample rate. The 250 Hz sample rate was used for all hydrophones (with the exception of one 1 kHz hydrophone in each of the Bransfield and Scotia arrays) because both array locations were primarily designed as passive seismic experiments and set to record low frequency earthquake-generated signals. These sample bands are also ideal for cryogenic sources and for recording the vocalizations of blue (*Balaenoptera musculus*) and fin (*B*. *physalus*) whales. The pre-amplifier was designed to equalize the spectra against typical ocean noise over the pass-band with an 8-pole anti-aliasing filter. A micro-processor controlled, temperature-correcting crystal oscillator with an average time drift of 1.95 s yr^-1^ provided accurate timing during the typical 1–2 year deployment duration. The electronics were housed in a titanium pressure case that was attached to a standard oceanographic mooring with anchor, acoustic release, mooring line, and a syntactic foam float to place the sensor at depths of 300–500 m within the ocean water column (Fig 2A and 2B).

### Sources of Ambient Sound

**Fig 3:**
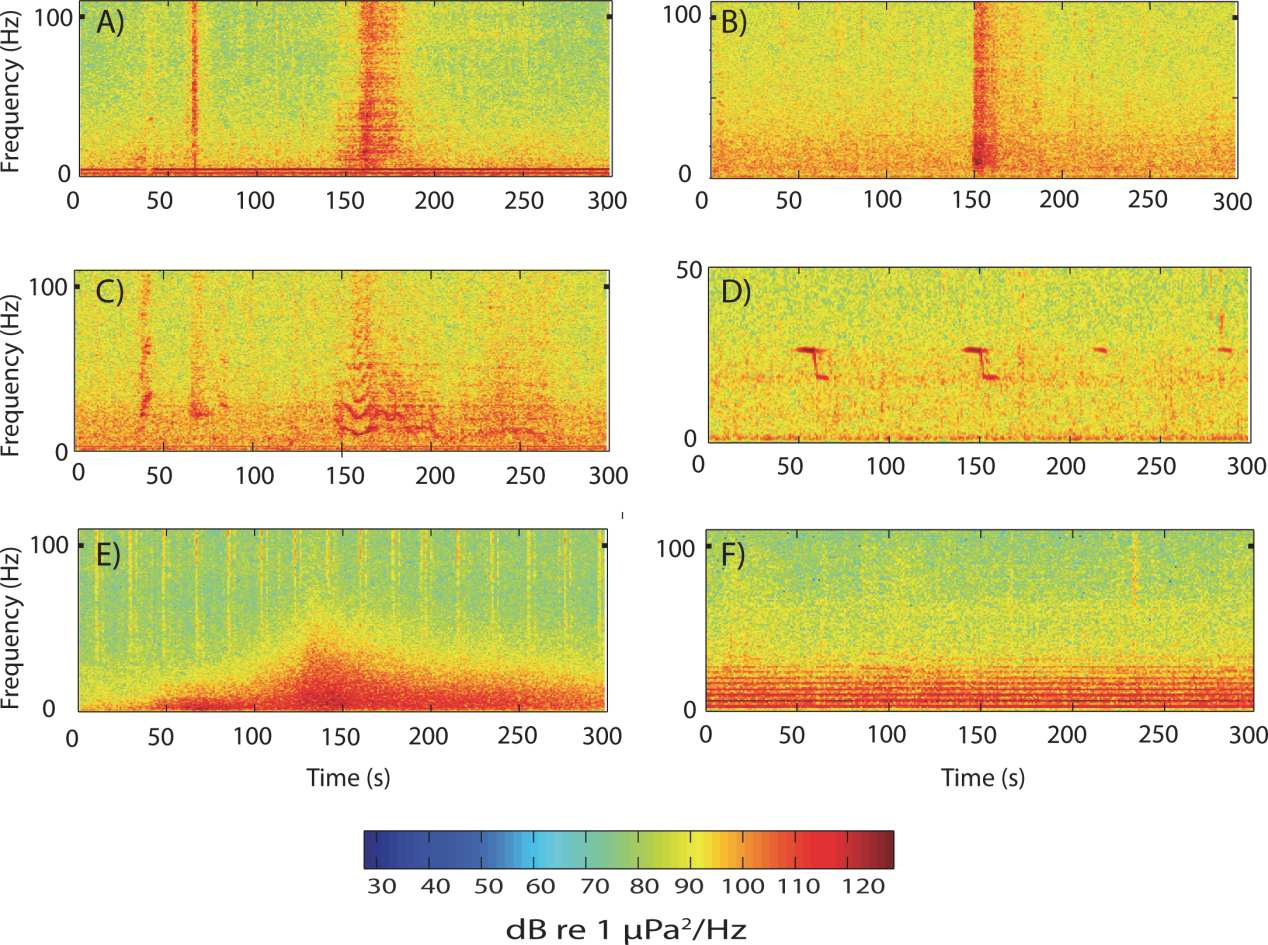
Spectrograms of the varied acoustics sources recorded by the Bransfield and Scotia Sea hydrophones. Panel (A) shows record of emergent, broadband, icequake acoustic arrival; (B) shows record of impulsive, short duration icequake signal, indicating that icequake may be closer to source, experiencing less attenuation than emergent record; (C) shows fundamental and harmonic overtones of iceberg tremor; (D) shows Antarctic blue whale vocalization; (E) is an earthquake (T-phase) signal packet with broadband airgun arrivals in background; and (F) is noise from a ship, generated by cavitation from the propeller. The sound energy level is roughly equivalent for all sources; however, each varies in prevalence through the year.

The Bransfield Strait and Scotia Sea hydrophone arrays were deployed in elliptical patterns to optimize recording and locating of low-frequency earthquakes from volcanic centers within and near both regions. Once the arrays were recovered, the utility of the data for analyzing cryogenic and other natural sources of sound was realized. Fig 3 shows the spectrograms of the most common acoustic signal sources recorded on the Bransfield Strait and Scotia Sea hydrophones, which vary from biological to geophysical to cryogenic. The signals that dominate the records (Fig 3A and 3B) are the broadband acoustic arrivals of icequakes from the break-up of icebergs and large blocks of sea-ice. Icequakes can be emergent (Fig 3A) or impulsive and short duration (Fig 3B) and it seems both signal types are likely due to source effects at the iceberg [13]. Fig 3C shows the fundamental frequency and harmonic overtones of tremor from a grounding iceberg. Fig 3D shows the very distinct Antarctic blue whale call, characterized by a repeating 28 Hz tone plus downsweep [10] and Fig 3E shows the acoustic coda of a seafloor earthquake (referred to as a “T-phase”) with broadband signals of an airgun in the background. Lastly, Fig 3F shows the characteristic sound created by a ship, generated by cavitation from the propeller.

### Hydrophone Location Accuracy

Accurate ocean sound-speed models and good azimuthal distribution of the autonomous hydrophone array relative to the Bransfield Strait and Scotia Sea allowed for well-constrained icequake locations (see [11] for detailed methods). A two-dimensional, nonlinear regression algorithm was used to minimize differences in observed and predicted arrival times at each hydrophone. Event location error is available as output from the regression algorithm covariance matrix [14], with the range in location error within the aperture of both arrays of ± 0.5–5 km in latitude and longitude at the 68% confidence interval [11,15]. Location error increases outside of the arrays, increasing to ± 10–20 km in latitude and longitude northward along the Antarctic Peninsula and toward the Scotia Island arc. The sharp onset, high-amplitude first arrival of the icequake acoustic signal packets was usually selected as the arrival time of the icequake for location purposes, since the icequakes are typically impulse functions generated by the instantaneous crack formed within the ice [11]. An acoustic magnitude, or source level (SL), was calculated for each icequake or grounding harmonic tremor (HT) event by removing the effects of attenuation and geometric spreading along the propagation path and the hydrophone instrument response [16]. This provides an estimate of the acoustic SL back propagated to a distance of 1 m from the source.

### Bransfield Strait and Scotia Sea Icequakes

The hydrophones recorded the relatively high-frequency, broadband arrivals of “icequakes” that result from cracking, collision, and breakup of icebergs, ice flows, and ice sheets within and along the periphery of the strait (e.g., Fig 2). Icequakes may be caused by thermal stresses, as well as physical deformation of the ice from wind, currents, and waves that can cause fracturing and colliding of ice blocks producing sound [5,6]. These external forcing factors result in shear failure of the ice crystal lattice, generating pressure waves that emanate into the water column. In high-latitude regions, the ocean temperature profile is nearly homogenous [17], and in response to rising pressure the sound velocity profile increases linearly with depth. This acoustic setting is known as a Polar half-channel, whereby sound propagates laterally through a series of sea surface reflections and upward turning refractions [18]. Thus, over the relatively short distances (~200–300 km), the waves propagate at mid-water depths with little seafloor interaction [11]. Once an estimate of the source location is known, the transmission loss along the ray path for each region can be calculated and an estimate can be made of the acoustic magnitude of the icequake [11,13]. The transmission loss estimate varies for the Bransfield Strait and Scotia Sea because of the difference in water depths for the two regions (~2000 m maximum depth in Bransfield Strait, versus ~4500 m in the Scotia Sea), which changes the amount and duration of the sea-surface, seafloor interaction of the propagating acoustic wave.

**Fig 4:**
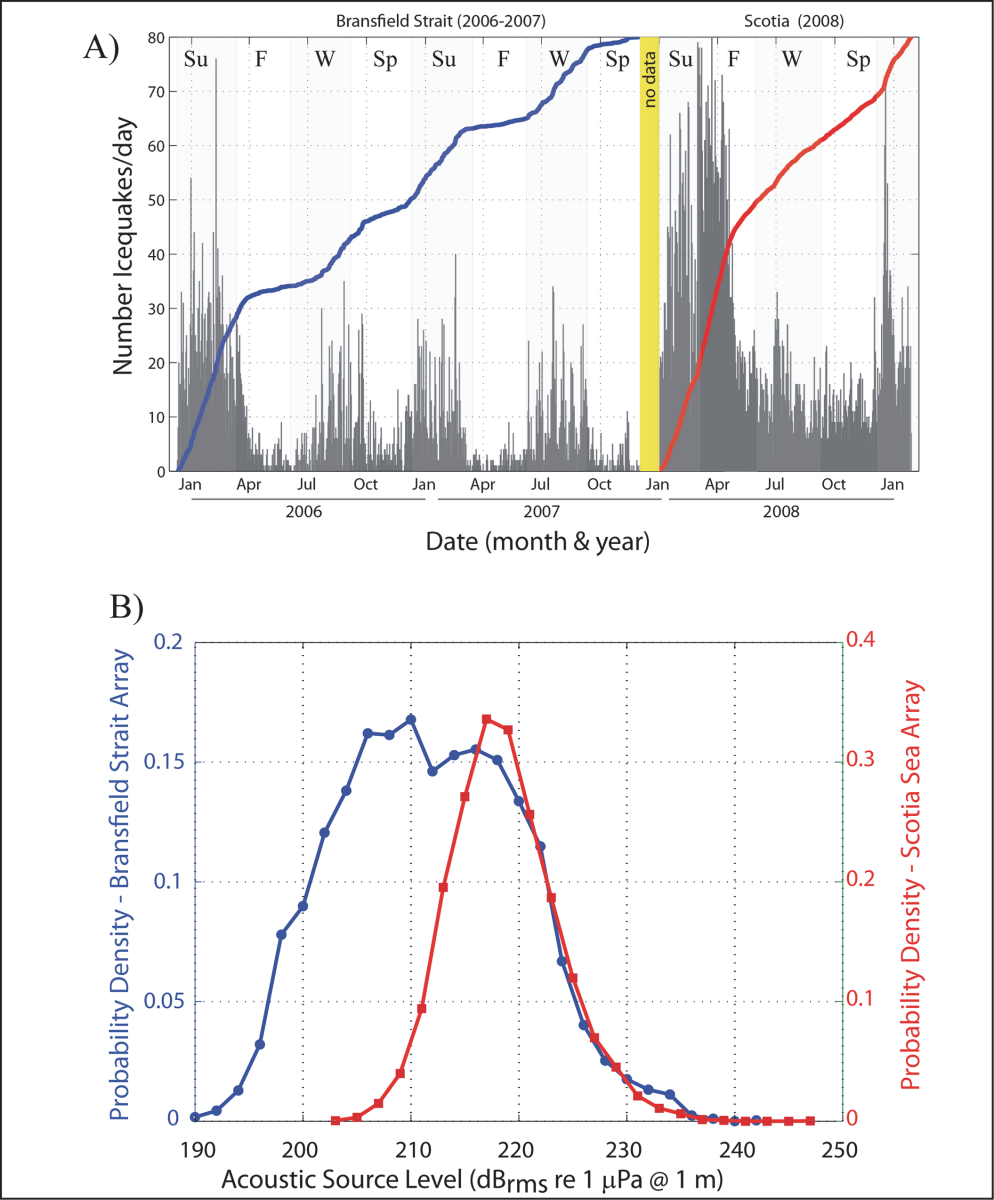
Histograms of iceqauke counts and source levels. (A) Number of icequakes per day recorded within the Bransfield Strait (left) and Scotia Sea (right). The Scotia Sea exhibits roughly four times the number of icequakes as observed in the Bransfield Strait. Bransfield icequakes show clear seasonal distribution, peaking roughly during the austral summer months and minimum during austral winter. Scotia icequakes also show seasonality, however peak in acitivty in early 2008 is due to breakup of A53a (Fig 5). For reference, gray background denotes begin and end times of seasons, with summer (Su), fall (F), winter (W) and spring (Sp) labeled. (B) Histogram showing estimates of Bransfield and Scotia icequakes source levels, which range between 190–242 and 203–247 dBrms re 1 μPa @ 1 m, respectively.

A total of 5925 and 9611 icequakes were located within the Bransfield Strait and Scotia Sea, respectively, using data from the hydrophone deployments during 2005–2009. Fig 1 shows the spatial distribution of icequakes located throughout both regions, while Fig 4A shows the histogram counts of the number of icequakes per day recorded. The Scotia Sea exhibited roughly 1.5 times as many icequakes over a shorter time period than in the Bransfield Strait. This difference in icequake counts is likely due to the presence of a large iceberg that traversed the Scotia Sea during the one-year recording period (more discussion on this below). Thus there are clear differences in environmental conditions between the two regions because the data were not recorded contemporaneously, and recored sound levels are not purely as a function of location. Source levels for Bransfield Strait icequakes were initially calculated using the maximum amplitude [11], whereas the source levels of Scotia Sea icequakes were calculated using the root-mean-square amplitudes within a time window during which 90% of the cumulative signal energy is received [13,19]. The two source level scales, however, are well correlated and so we have corrected all data to root-mean-square amplitudes using the empirical relationship between them. The Bransfield and Scotia icequakes exhibited acoustic energy (source) levels that ranged between 190–242 and 203–247 dBrms re 1 μPa @ 1 m, respectively (Fig 4B). The larger relative proportion of small source level events detected by the Bransfield array is consistent with its much smaller aperture, relative to the Scotia Sea array (Fig 1).

The icequake locations in the Bransfield Strait during the three-year recording period paralleled the coast of the Antarctic Peninsula (Fig 1B), and were distributed throughout the Strait. The icequakes mainly clustered along the southwestern portion of the basin, which is the shallower part of the basin as it shoals to the southwest toward Deception Island, as well as along the coastline of the South Shetland Islands. The icequake locations also clustered in two to three lobes that trended toward the Antarctic Peninsula along the northeast part of the Bransfield Strait. Notably, they appeared to parallel the trends of submarine canyons, suggesting that sea ice may accumulate in areas of glacial outflow [11]. A similar correlation between ice-tremor and outlet glaciers has been noted for the Wilkes Land coast [20]. Due to location error associated with the hydrophone station geometry, locations derived for events located outside of the array aperture will tend to cluster along common travel-time hyperbole that point toward the true source location [21]. The Scotia Sea icequakes are also widely distributed throughout the region, and similarly cluster along lines directed from the hydrophone array toward the South Sandwich Island group (Fig 1). Thus, many of the icequakes located using the Scotia Sea array are sourced at or near the islands, potentially coming from land glaciers with ocean entry [22] or resulting from the grounding of icebergs along the island shores. Dozens of Scotia Sea icequakes also clustered along the path of iceberg A53a as it passed through the hydrophone array moving east toward South Georgia Island. We interpret these clusters of icequakes as likely sourced from A53a, representing fracturing of the berg as it sailed through warmer ocean water to its eventual demise. Moreover, many icequakes are located along the continental shelf of South Georgia Island, which may be the result of the impact of the iceberg keel with the seafloor (Figs 1B and 2).

Counts of icequakes varied regularly with season in both the Bransfield Strait and Scotia Sea, where icequakes peaked during the austral summer months and were at a minimum during fall and spring (Fig 4A). Interestingly, the number of icequakes also showed a distinct increase in both regions by the end of austral winter, early spring (September–October), which was likely caused by fracturing of large blocks of sea ice (icebergs) as air temperatures began to increase. The icequakes decreased again in the Bransfield Strait during early summer 2006 (December) before increasing again in late summer and fall. It is not clear what caused the low icequake count during early summer; it could be that calmer weather (low wind speeds and wave heights) leads to reduced ice breakup during this time as has been observed in the Arctic [7].

The large peak in icequake counts in the first few months of the Scotia Sea deployment (late 2007–early 2008) is due to the presence and breakup of the large iceberg A53a in the region (more detail is provided on A53a in the following sections). The breakup of A53a as it sailed past South Georgia Island is the main source of icequakes in the Scotia Sea during the 2007–2008 hydrophone deployment. The second peak in icequake activity during December 2008 is from icequake sources outside of the array, located several array apertures to the north and east of South Georgia. Since A53a had left the area by then, the renewal of icequake activity was likely due to the entry of additional large sea ice into the region.

Auto-correlation of icequake activity indicates that the correlation has a maximum value at a lag of 207.3 days, with other peaks at 103.6 and 310.9 days [11]. This is consistent with an approximately 6-month seasonal periodicity, which again likely reflects variable thermal and wind stress conditions during the annual freeze-thaw cycle. The possible correlation of Bransfield Strait and Scotia Sea icequakes with ocean and solid Earth tidal stresses and sea-surface tidal height (e.g., [23]) also was investigated; however, no statistically significant correlation was found between tides and icequakes.

### Acoustic Signals from Iceberg A53a

Several hundred individual iceberg harmonic tremor (IHT) sounds were recorded on both hydrophone data sets. IHT are seismic and hydroacoustic signals associated with Antarctic icebergs, both adrift and aground, that have only been identified within the last decade [24,25]. The signatures of IHT are long duration (up to several hours), time-variable fundamental frequency that can range from < 10 Hz to over 40 Hz, with multiple overtones (Fig 2B). IHT has been observed tens to thousands of kilometers from the presumed source as hydroacoustic waves and locally converted seismic waves, on islands in the equatorial Pacific, mainland Antarctic, and Indian Ocean (e.g., [20,24,26]). Although there are numerous IHT events in the hydrophone data from both regions, they occur infrequently in comparison to icequakes and total less than 2% of the number of icequakes observed during the same recording period.

During April to June 2007, the iceberg A53a (~55 × 20 km) drifted out of the Weddell Sea and through the Bransfield Strait. Although the sounds produced by this iceberg were briefly summarized by Dziak et al. [27], the contribution of the sound energy release from this iceberg to the overall sound field is discussed here in order to consider all acoustic sources. Hydrophones first detected IHT from A53a when it impacted a 124 m deep shoal, causing the iceberg to rotate 192°, and began generating six days of continuous IHT as it ground across the seafloor (Fig 1A, position 1). The iceberg then entered the Bransfield Strait, where it became fixed and began to pinwheel over a 265 m deep shoal (Fig 1A, position 2). The IHTs became shorter in duration (40–60 s) in this area. The tremor fundamental frequencies, overtone spacing, and signal length are a direct function of the size and duration of the ice-seafloor contact and the speed of the iceberg as it is driven by wind and ocean currents [25].

Analogies to models of tectonic and volcanic tremor suggest three possible IHT source mechanisms: (1) frictional processes, where the iceberg tremors are generated by periodic, discrete stick-slip bursts caused by contact of the moving iceberg with the seafloor or other icebergs [25], (2) movements of water through englacial conduits [20,24,26,28], and (3) excitation of iceberg rift reservoir modes, induced by hydrodynamic instability of ocean currents moving past openings where rifts intersect the edge of the iceberg [29]. At the two locations in the Bransfield Strait where IHT was detected (Fig 1A), the seafloor is much shallower than at other spots along the iceberg’s track within the Drake Passage and Scotia Sea. Thus it seems likely that the iceberg tremors from A53a are being generated by stick-slip contact of the moving iceberg with the seafloor rather than a resonant vibration phenomena.

**Fig 5:**
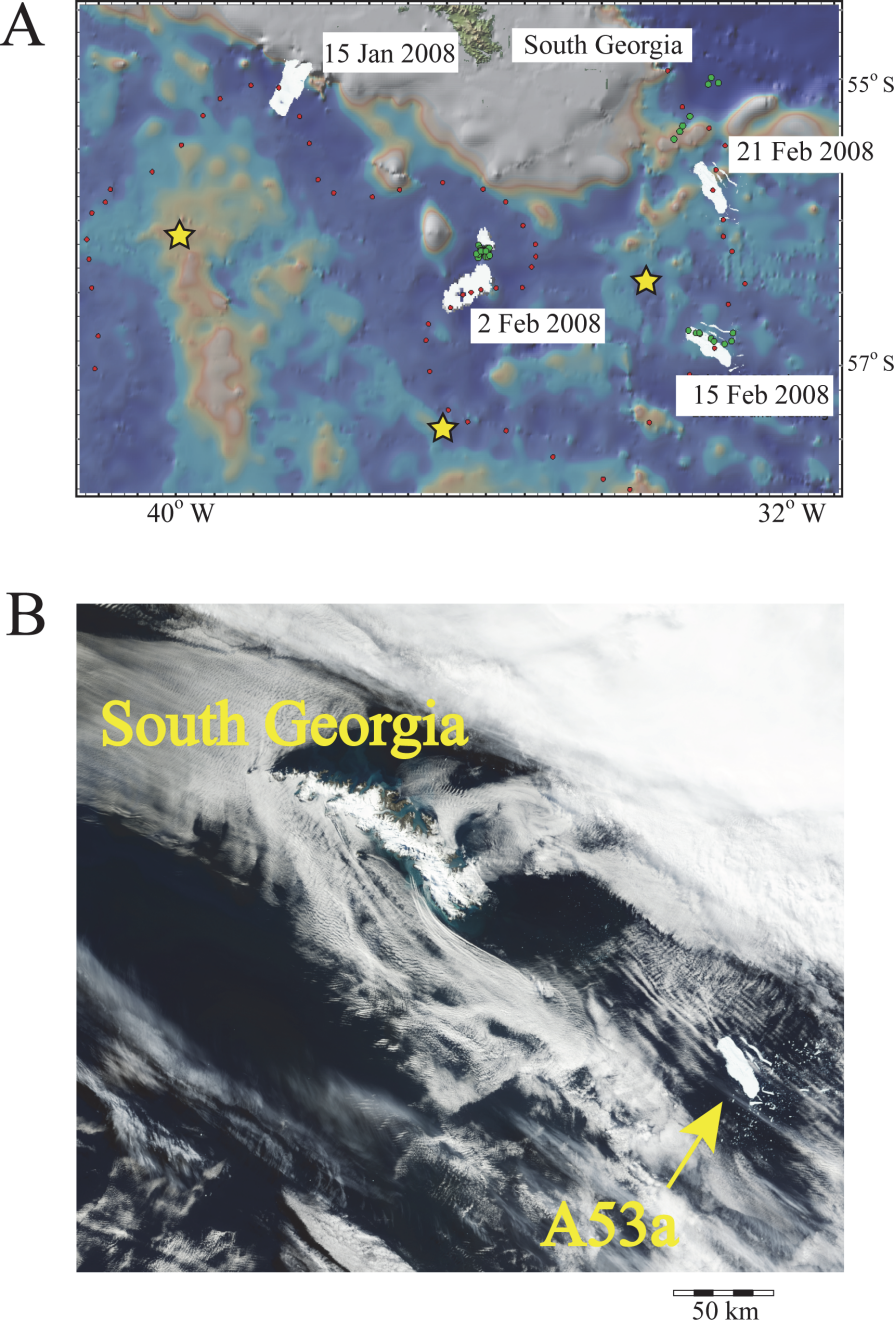
Satellite image and breakup of iceberg A53a in Scotia Sea near South Georgia Island. (A) Red dots show daily position of A53a as it rounded southeast tip of South Georgia. The dates of four positions of A53a are shown. The images of A53a are polygon representations made based on satellite images [50]. Satellite bathymetry shown is from Sandwell and Smith [49] and displayed using Geomapapp (http://www.geomapapp.org/). The first major breakup event occurs on 2 February 2008. Green dots show hydrophone derived locations of icequakes associated with disintegration of iceberg. Yellows stars are hydrophone locations. Scatter of icequakes on 21 February likely due to iceberg being outside array aperture. (B) Satellite image showing smaller scale views of disintegration of A53a as it rounded South Georgia following major breakup events on 2–21 February 2008[50]

The iceberg then drifted north during July 2007 into the Scotia Sea and began to melt [30], and within two months it had broken up and was no longer being tracked. Large fractures could be seen in A53a along the full length of the iceberg (e.g. Fig 5A and 5B). The disintegration that occurred within the aperture of the hydrophone array produced the large number of icequakes south of South Georgia Island seen in the icequake counts in February–March 2008 (Fig 4), and allowed for the unusual observation of an iceberg breaking apart while adrift [27]. These open ocean "calving sound" icequakes have the same signal characteristics discussed earlier (short-duration, broadband signals), generated by ice cracking and crack propagation (similar to earthquake processes) and clearly distinct from the IHT sounds [31]. Moreover, several icequake clusters were observed near the shelf break of South Georgia Island, likely representing where the iceberg's keel impacted the shelf. Dziak et al. [27] estimated the energy flux density (EFD) of a 20-minute long sequence of icequakes from A53a as it broke apart; the sequence was observed via satellite (Fig 5A). The EFD estimate was ~252 dB-μPa^2^-sec, which is equivalent to the energy release from dozens of individual icequakes and provides an example of the significant contribution that the breakup of these icebergs can have to the ambient sound field.

### Long-term Ambient Sound Sources and Levels

**Fig 6:**
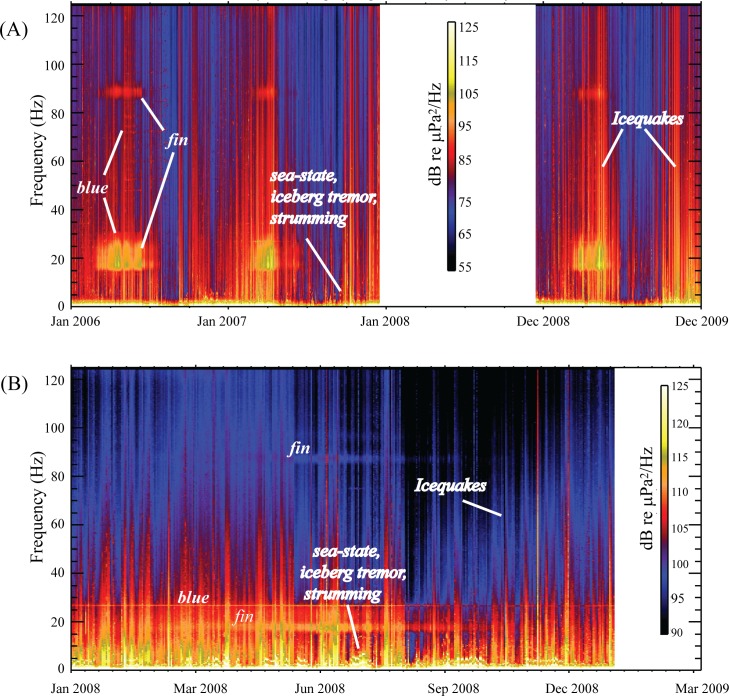
Spectrograms of acoustic power spectral at the Bransfield Strait (A) and Scotia Sea (B). Spectrograms are calculated from 2 second FFT windows with each vertical time slice representing the cumulative energy over a 1 day interval. Near-constant, broad-band, short-duration impulsive signals are likely sounds from wind-driven waves and ice breakup. Clustered energy in the 15–25 and 89 Hz bands are fin whale calls. Blue whale calls are the steady tone at 27 Hz. The several examples of fundamental and overtone energy <10 Hz in both spectra are interpreted to be from a combination of sources, including broadband energy created by sea-state (storms, waves, and wind), iceberg grounding tremor, and tonal “strumming” of mooring line caused by fast moving ocean currents. White areas represent times with no data.

**Fig 7:**
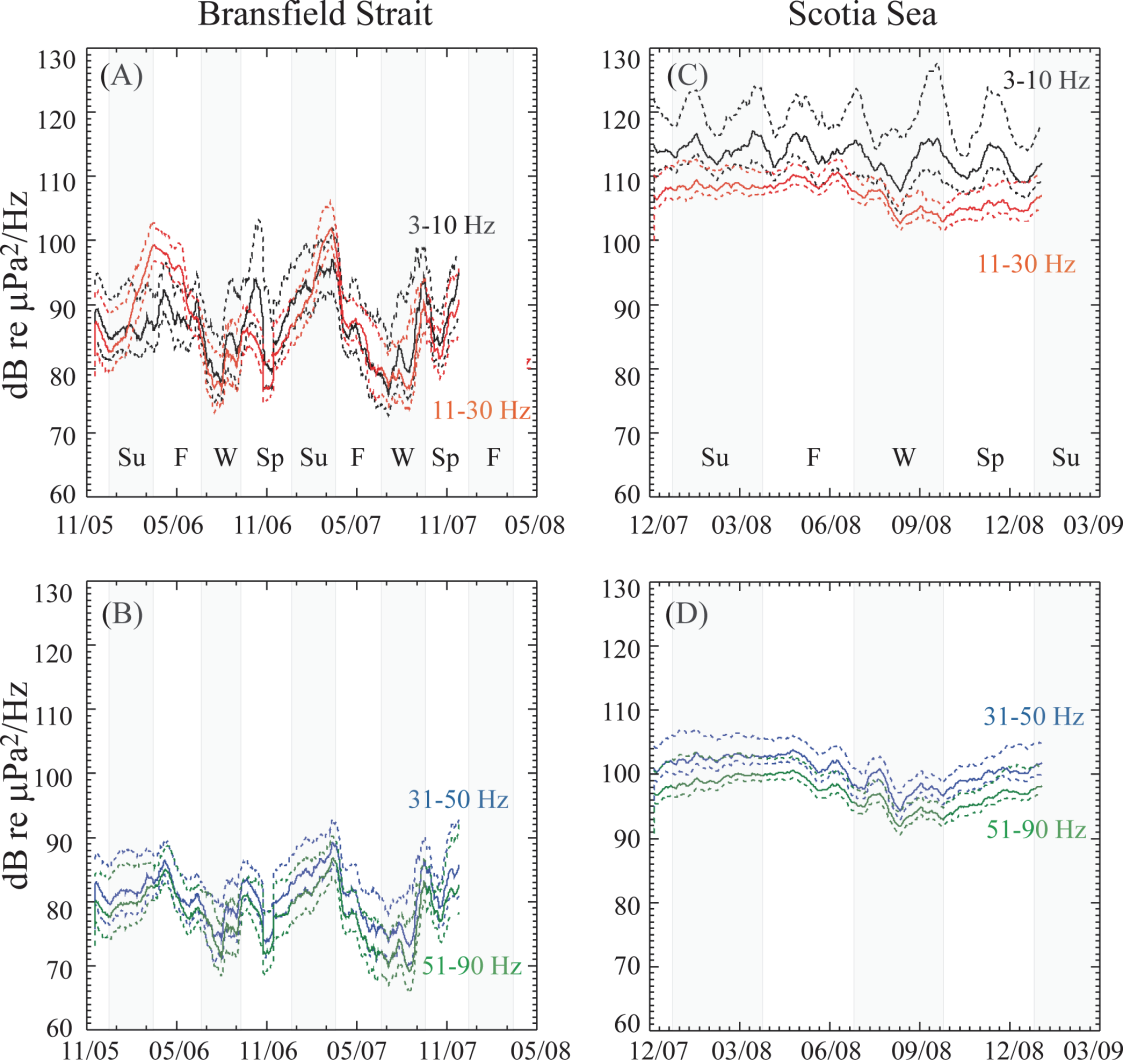
Diagram of selected percentiles from the cumulative distribution of spectral energy (daily average). Spectrograms shown are hydrophone M2 in Bransfield Strait (A-B; 2005–2007) and M4 in Scotia Sea (C-D; 2008–2009). The noise levels are separated into select frequency bands to examine the influence of different sound sources on overall levels. Solid line shows the dB levels of 50% of the cumulative spectral energy in the given frequency band; lower dashed line shows the dB level of 5%; and upper dashed line shows the dB level of 95% of the cumulative spectral energy. Gray background marks begin and end of seasons as in Fig 4.

Long-term spectrograms of the acoustic power spectral density and the daily average sound levels recorded on hydrophone M2 in the Bransfield Strait and M4 in the Scotia Sea are shown in Figs 6 and 7. Since the Bransfield Strait array was a three-year deployment as compared to a one-year deployment in the Scotia Sea, the signals in Fig 6 appear much more compressed in the Bransfield Strait spectra. For consistency in comparison, the frequency-dependent hydrophone instrument response was removed from both spectra prior to display.

From Fig 6, overall noise levels appear to be higher in the Scotia Sea than the Bransfield Strait. The near-constant, broad-band, short-duration impulsive signals present in both spectra in Fig 6 during the austral summer to fall (December to June) are icequakes, or ice-breakup sounds, likely due to increased summer temperatures and wind-driven ocean waves. There is also continuous, low-frequency energy present in both regions. This energy is focused under 5 Hz in the Bransfield Strait, and under 10 Hz in the Scotia Sea. During several periods of time, which can last from weeks to months, the energy develops into tremor-like signals with a fundamental at 1–3 Hz and several overtones. We interpret this low energy to be from a combination of sources, including broadband energy created by sea-state (storms, waves, and wind), minutes-to-hours long periods of iceberg grounding tremor, and periods of tonal “strumming” caused by fast moving ocean currents that make mooring line vibrate. Although the icequakes show a clear seasonal variation, this low-frequency energy remains present throughout the year. This is consistent with the idea that this energy is caused by currents, storms, and grounding icebergs that are present year-round in the Southern Ocean.

The difference in sound levels between the Scotia Sea and the Bransfield Strait can be more clearly seen in the comparison of sound levels between the two regions in Fig 7, which shows selected percentiles of the long-term, cumulative distribution of spectral energy (daily average) recorded on hydrophones M2 (Bransfield Strait) and M4 (Scotia Sea). The noise levels were separated into four distinct frequency bands to examine the influence of different sound sources on overall levels, with the 3–10 Hz band for iceberg tremor, and to avoid potential mooring line strumming noise (from fast ocean currents), which is <3 Hz, 11–30 Hz for blue and fin whales, 31–50 Hz for icequakes, and 51–90 Hz for a combination of icequakes and fin whale pulses. The daily sound level (dB) distribution over time in each of two frequency bands is shown in the four panels of Fig 7. The Scotia Sea ambient sound levels were not overlain with the Bransfield Strait data because the data sets are not time synchronous. Sound levels are from10 to 20 dB lower in the Bransfield Strait than the Scotia Sea in all frequency bands. Nonetheless, the Scotia Sea noise levels show similar seasonal variations as the Bransfield Strait. For example, the 51–90 Hz bands have peak energy during austral fall-winter (May–June), likely due to increasing wind speeds, increasing sea-ice cover and contributions from peak blue and fin whale vocalizations during this time. The downward trend in noise levels leading to a minimum during August (late austral winter), when ice cover and wind speeds should be a maximum but whale vocalizations are low, also mirrors the minimum levels observed in the Bransfield Strait. In comparison, Arctic noise studies showed a similar seasonal variation, where months with significant ice coverage exhibited low noise levels, months with both ice coverage and low winds exhibited even lower noise levels, and months with ice-free open water had the highest noise levels [7]. Roth et al. [7] show ambient noise increases with wind speed by ~1 dB/m/s for open water. Thus, we interpret this decrease in sound level during winter as being due to a similar effect, where sea ice cover correlates with reduced ambient noise (perhaps through reduced sea surface wave action). In contrast, all frequency bands show a significant rise in noise levels from spring to early summer (September–December), consistent with Arctic observations where ice-free open water has the highest ambient noise values [7] as well as the Bering Sea where environmental sound levels are louder during non-winter months with the soundscape dominated by wind and precipitation in the fall, and a combination of marine mammals and ice in the spring [32,33].

Fig 7 also appears to show that blue and fin whale vocalizations may exhibit different sound levels in the two regions (in the 11–30 Hz band). However, this difference may be caused by a contrast in background noise levels between the two environments, a difference in the distance the whales are from the recording hydrophone, or even differences in the distribution of the animals. A limitation of omni-directional hydrophones is that it is not trivial to distinguish between a few, very vocal animals near the hydrophone or the summed energy of numerous animals further from the hydrophone. Because the Scotia Sea hydrophones are in a more open, deep water environment, the whale vocalization energy received could be from distant blue and fin whales via the deep sound channel.

Seafloor earthquakes also contribute to the overall noise spectrum within both the Bransfield Strait [11] and the Scotia Sea [17]. The majority of acoustic energy from earthquakes is <25 Hz, which overlaps with the lower icequake band and is in the frequency range of IHT observed in both regions. The number of earthquakes detected using T-phases recorded from the Bransfield Strait and Scotia Sea on the hydrophone arrays was 3900 and 1055, respectively, and therefore represents ~66% and 11% of the total number of icequakes for each region. This suggests earthquakes in the Bransfield Strait may contribute significantly to the ambient sound field, while in the Scotia Sea, the impact of earthquake noise is more likely a minor component. However, since earthquakes should not show a seasonal fluctuation in counts, as icequakes do, and are more randomly distributed [11], our interpretation is that any seasonal pattern observed in the long-term noise spectrum is likely derived mainly from icequake energy. Given that earthquake rates are significantly higher in the Bransfield Strait than in the Scotia Sea, yet ambient sound levels appear relatively higher in the Scotia Sea, earthquake sound energy may be a relatively minor contribution to the sound field in both regions.

The acoustic energy focused in the 15–28 and 89 Hz bands (Fig 6A and 6B) is from fin whale calls [8,9]. The steady signal within the 28 Hz band (Figs 3D and 6) is the vocalizations of Antarctic blue whales [8,10,34] that overlap seasonally with fin whale calls. The blue whale vocalizations (most easily seen in the Scotia Sea data) are characterized by a long-duration (~10 sec) 28 Hz tone, sometimes followed by a downsweep to another tone at 19–20 Hz [10] (Fig 3D). However, the long-term spectrogram (Fig 6) shows blue whale signal energy predominantly focused in the 28 Hz band with little downsweep energy apparent. Blue whales can generate overtones up to 80 Hz, which are occasionally seen in the spectra but were primarily apparent during February 2006 in the Bransfield data. Consistent with the findings of Širović et al. [8], fin whale vocalizations showed a clear seasonality, with a maximum during the austral fall-winter months, following the peak in icequake activity. Blue whale calls were, however, present nearly year-round, which is also consistent with the previous findings of Širović et al. [8]. As can be seen from Fig 6, the vocalizations of these whales are a significant, nearly continuous, component to the ambient sound field in this region. Moreover, the blue whale vocalization energy levels in the 28 Hz band appear to be fairly similar between the Scotia and Bransfield, both falling within in the 115–120 dB range. These levels are consistent with received levels of blue and fin whale vocalizations published by Širović et al. [8].

**Fig 8:**
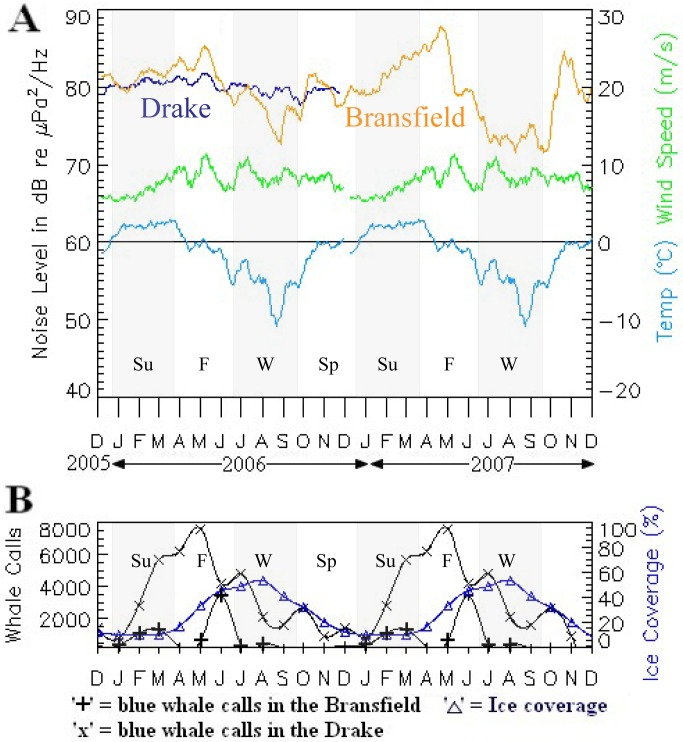
Bransfield ambient noise levels compared to wind, temperature, ice cover and blue whale vocalizations. (A) Examples of ambient noise levels (averaged over 51–90 Hz) in Bransfield Strait (yellow) and Drake Passage (light blue), air temperature (dark blue), and wind speed (green) from December 2005 to December 2007. Wind speed and air temperature were measured at King Sejong Station, on King George Island (red dot on Fig 1B). Gray background marks begin and end of seasons as in Figs 4 and 7. (B) Shows seasonal variation of 27–28 Hz energy (blue whale occurrence) in Bransfield (yellow cross), Drake Passage (blue x), and sea-ice coverage (purple triangle). Sea-ice concentration taken from National Snow and Ice Data Center [51]. High overall noise levels correlate with lowering temperatures, average winds, low ice cover, but high whale vocalization energy.

Fig 8A shows ambient noise levels averaged over 51–90 Hz from one hydrophone in the Bransfield Strait and one in the Drake Passage. We chose this band to minimize the blue and fin whale call energy contribution, in an attempt to highlight the ambient noise levels due to ice, meteorological, and geophysical (non-biological) sources. One caveat is that this band still includes the 89 Hz fin whale high pulse, which will influence ambient sound levels during peak calling months. The sound averages from the Bransfield Strait and Drake Passage were then compared to the daily record of air temperature and wind speeds measured at King George Island, located between the Bransfield Strait and Drake Passage, illustrating the relationship between environmental factors and noise levels. Interestingly, ambient noise levels were lowest in the Bransfield Strait during the lowest annual temperatures, high ice cover of the austral winter months. The low noise during this period is again consistent with Arctic observations, where sea ice cover correlates with reduced ambient noise [7]. Noise levels are highest in the Bransfield Strait during austral fall, presumably when wind and wave heights are beginning their seasonal increase yet ice cover is not a maximum.

The Drake Passage does not show as large a variation in noise levels as the Bransfield Strait, possibly due to its lack of extensive ice cover. However, the highest noise levels in both the Bransfield Strait and Drake Passage correlate with the times of the highest recorded wind speeds during the austral fall-winter (May–September). This is also consistent with observations of increasing wind speeds leading to increasing noise levels [7]. There is evidence that wind induces high noise conditions by increasing wave heights [4,35], both of which will increase through the fall and winter months. The reduced noise levels in winter, however, may reflect the increase in ice cover through the winter months in these regions, which may in turn offset noise increases due to seasonally increased wind speeds.

We next looked at the distribution of blue whale vocalization energy over time (Fig 8B) as a means to gauge the ecosystem response and/or interrelation to other environmental factors. The peak in noise levels seen in the Bransfield-Drake data during the austral late fall correlates with a peak in blue whale vocalizations within the Bransfield Strait and the Drake Passage (Fig 8B). The seasonal presence and relative abundance of blue whale populations are inferred from the mean monthly acoustic energy level detected on hydrophones in the 27–28 Hz band. Here we assume an increase in calling level is indicative of an increase in the number of animals in the area. If this assumption is true, blue whales have a peak presence in the Drake Passage from February to May in 2006 (only one year of data), while blue whale calls in the Bransfield Strait peak in March–April in 2006 and February–March in 2007. Blue vocalization energy is at a maximum in Bransfield Strait and Drake Passage when sea ice coverage is at a minimum (Fig 8B). Thus, the blue whale call energy is at a maximum in the Drake Passage and Bransfield Strait when overall ambient noise levels are at a maximum (Fig 8A) and sea-ice cover in the Bransfield is at a minimum (Fig 8B). A slight increase in blue whale energy was detected during a peak in ice cover during August–September 2006, but this increase was still well below peak values seen at other times and correlated with minimum ambient noise energy in the Drake Passage. Thus, it appears that blue whale occurrence reaches a maximum in the early austral fall months in both areas, but is relatively low just before the peak freeze during winter when sea-ice cover is heaviest. Nevertheless, blue whales maintain a year-round presence in the region as observed in previous studies [8,9].

## Discussion

### Spatio-Temporal Icequake Patterns

Overall, hydroacoustic data show that the Bransfield Strait and Scotia Sea are areas of dynamic sea-ice and ice berg activity based on the thousands of icequakes and other cryogenic sound sources recorded in both areas. The icequakes in both regions show a strong seasonal variability, reflecting the freeze-thaw cycle with most icequakes being detected during the austral summer months likely due to increased thermal stress, and are a minimum during early winter (Fig 4). Interestingly, the number of icequakes begins to increase again in both regions during the late austral winter (August to September), somewhat in contrast to the low ambient noise levels during winter seen in the long-term spectra. We speculate this winter increase in icequakes may be caused by fracturing of ice cover due to either wind and wave stress from increased storm activity, or perhaps cracking from hard freezing as the lowest air temperatures occur.

Given the large number of icequakes and their high (>190 dB) source levels, the noise from icequakes is clearly a significant and sustained source of ocean sound in the Bransfield Strait and Scotia Sea. For comparison, the maximum icequake source levels (≥220 dB) are equivalent in acoustic energy to moderate to large magnitude (~5 m_b_) earthquakes [11]. The greater number of icequakes recorded during one year in the Scotia Sea, compared to three years in the Bransfield was likely caused by the presence of iceberg A53a in the Scotia. Thus the Scotia Sea icequake record presented here likely represents an atypical year of ice-induced environmental sound, and may normally be at much lower level.

The icequake locations along the east side of Bransfield Strait show a separation into lobes that trend toward the Antarctic Peninsula (Fig 1B). We interpret these icequake clusters as likely being caused by impact of icebergs with the seafloor as they are calved from glacier fronts and are channeled along the shallow portions of submarine canyons toward the open ocean [11]. Moreover, the majority of the icequakes near the Antarctic Peninsula are located at depths less than the 500 m bathymetric contour, consistent with retreat of the ice fronts from the 400 m ground line since the Last Glacial Maximum [36].

The icequake locations in the Scotia Sea (Fig 1A) are distributed throughout the entire region. Because of the prevailing west to east wind and ocean currents in the Drake Passage, all of the icebergs and sea-ice that originate in the western Weddell Sea and drift northward are forced northeastward, with track-lines that pass within and through the Scotia Sea on their way to the South Atlantic (e.g., [37]). Thus, the Scotia Sea is an area that experiences a large volume of ice subjected to higher physical stress from wind and wave action, and leading to rapid ice fracturing in the relatively warmer ocean water.

There is also a clear regional clustering of icequakes in the Scotia Sea. Icequakes concentrate along the path of iceberg A53a as well at several locations along the continental shelf off South Georgia Island. The clusters of icequakes associated with the path of A53a were likely caused by the disintegration of the berg as it sailed northeastward. Given large Antarctic tabular icebergs can exhibit keel depths up to 750 m below the surface, with the majority reaching no deeper than 400 [38], we interpret the icequakes along the South Georgia shelf likely represent points where the iceberg keel impacted the seafloor, causing icequakes within the berg. The icequake counts (Fig 4) peaked during January–March 2008, likely due to the increased rate of disintegration as the iceberg rounded South Georgia, impacted the shelf and entered warmer waters at lower latitudes. We also observe that locations of icequakes from several small arcs that trend toward the South Sandwich Islands in the eastern Scotia Sea. These arcs are the locations of icequakes that are actually sourced from icebergs near ocean front glaciers on the islands, but the locations fall along common travel-time hyperbolae because they are outside of the array aperture and locations are not well-constrained. Another possiblity is that because icebergs typically move eastward in the Scotia Sea, these icequakes are from icebergs that have moved toward and grounded near the Sandwich Islands.

### Ambient Noise Levels

Long-term ambient sound levels appear to be significantly higher in the Scotia Sea than the Bransfield Strait over the frequency band sampled here (3–90 Hz, Fig 7). Moreover, the 3–10 Hz and 11–30 Hz frequency bands from the Scotia Sea exhibited the highest ambient noise levels over the seasons, but were especially high during the austral spring-summer months from September to December 2008 (Fig 7C), likely due to low frequency energy from wind, waves, iceberg tremor, and icequakes during this time (Fig 6B). By September to December 2008, iceberg A53a had exited the Scotia Sea and sailed well north of South Georgia Island, thus the noise level from icequakes during this time should be considered typical of the region. The direct comparison of the multi–year dataset at Bransfield Strait and the Scotia Sea is constrained by the limited amount of temporal overlap between the datasets (~1 month) and that the Scotia Sea icequake counts may be atypical because of the presence of A53a iceberg in early 2008. These two factors may account for some of the disparity in overall sound levels between the Bransfield Strait and Scotia Sea. However, the whale calls are also a significant part of the sound levels observed in the two regions, and likely contribute to the clear seasonal differences in sound levels observed in both regions discussed below.

The 3–10 and 11–30 Hz bands in both the Scotia Sea and Bransfield Strait also exhibit the highest ambient sound levels for every season throughout the year as compared to other frequency bands. However, as Fig 6 shows, the icequakes and whales exhibit peak energy from austral summer to mid-winter (December to September), with lows in late winter–early spring. This strong seasonality can be seen in the sound level percentile diagrams with peaks in 3–10 and 11–30 Hz bands during these months. It seems reasonable to assume the icequakes peak during summer-fall because temperatures increase and icebergs and sea-ice of all scales breaks apart. Indeed for the Scotia data, this peak likely reflects the increased icequakes from the grounding of A53a on the South Georgia shelf. However, the summer-fall is also a peak in the presence of baleen whale sound, whose vocalizing contributes to the ambient sound levels. During winter, wind speeds increase, temperatures decrease; however, ice cover also increases (Fig 8B), which may keep noise levels reduced despite increasing wind speeds. Moreover, whales are not present in high numbers. Therefore ambient noise levels are relatively low during the winter-early spring seasons.

The Scotia Sea and Bransfield Strait also show seasonal variability in the 31–50 and 51–90 Hz bands, with peak energy during austral summer to late fall (December to May). This again is likely due to an increase in sea-ice breakup from seasonal increases in ocean and air temperatures, the presence of A53a in the Scotia in early 2008, contributions from peak blue and fin whale vocalizations during this time, and possibly the interaction of wind with more open water (in the summer) leading to increased wave heights [7]. The downward trend in noise levels leading to a minimum during late austral winter (August) also mirrors the minimum levels observed in the Bransfield Strait, likely due to more ice coverage [7].

Even though the seasonal variability observed in the cumulative sound levels is similar for all frequency bands (Fig 7), the 31–90 Hz band exhibits significantly lower sound levels than the other bands in both the Scotia and Bransfield. This is likely due to the absence of whale vocalization energy in this band, as well as reduction in the energy contributed from iceberg tremor and icequakes. Although icequakes are broadband signals and have energy in the 31–90 Hz range, much of the icequake energy is centered in the 3–30 Hz band, and do not contribute much energy to the higher bands.

Overall, the distributions shown in Fig 7 indicate sound levels show similar seasonal variation in the Scotia Sea and Bransfield Strait even though there is little time overlap in the datasets. This suggests that noise generating processes in the two regions are similar, year to year, despite their geographic and water-depth differences. However, the sound levels can be from 10 to 20 dB higher in the Scotia Sea than the Bransfield Strait in all frequency bands. We speculate that this is because long-distance, low-frequency sound cannot enter the relatively shallow waters of the Bransfield Strait as it is blocked by the Antarctic Peninsula and offshore islands. Thus regional sources of sound do not contribute low-frequency energy (for this study, ≤ 90 Hz) to the ambient sound field in the Bransfield Strait, keeping the levels in these bands relatively low. In contrast, the Scotia Sea hydrophones are relatively more exposed to distant sources from elsewhere in the Southern Ocean, causing low frequency sound levels to be relatively high since they are a combination of local and regional sound sources.

**Fig 9:**
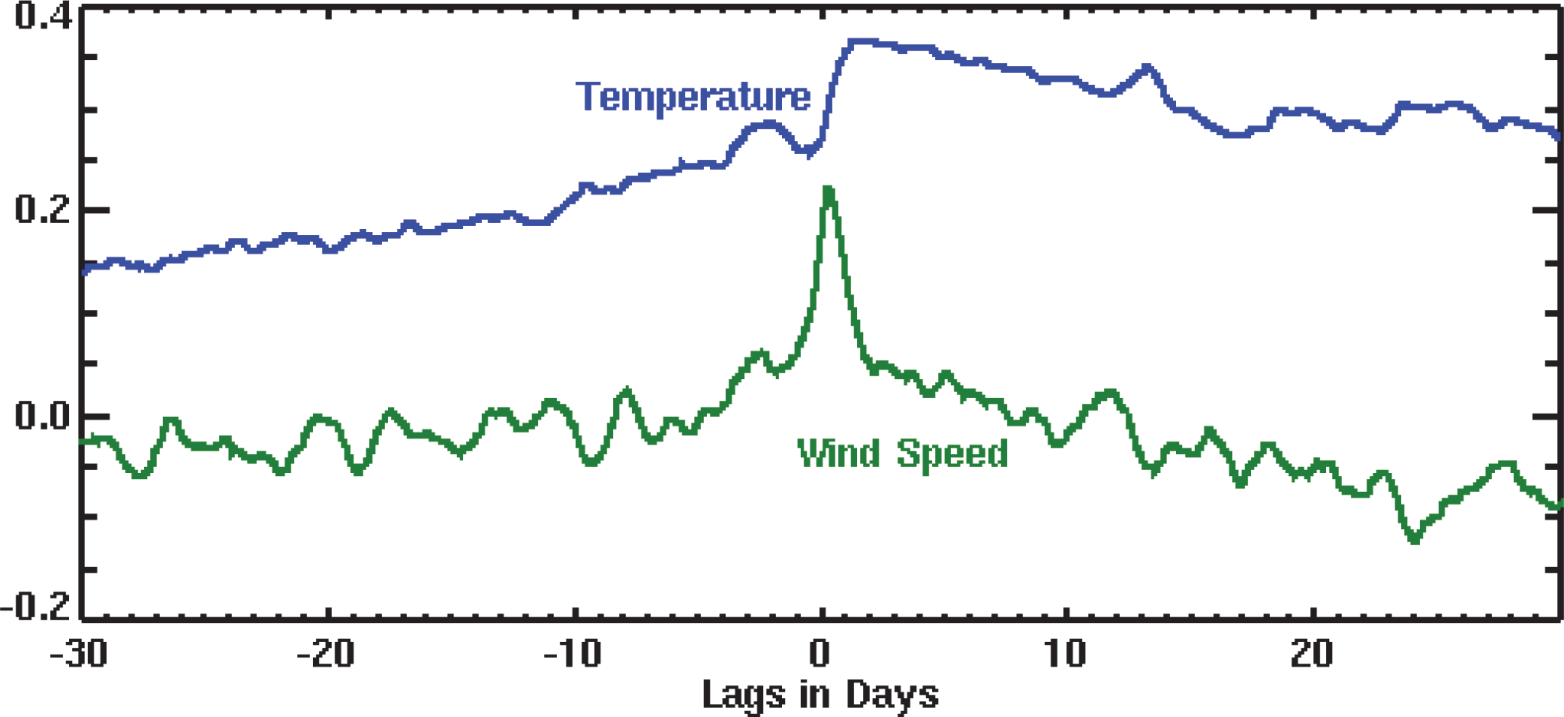
Cross-correlation functions of mean noise level in Bransfield Strait with temperature and wind speeds measured at King Sejong Station on King George Island. Noise levels were recorded on hydrophone deployed in the Bransfield Strait in the 51–90 Hz band. Noise levels show a direct correlation with wind speed, whereas noise levels lag an increase in air temperature by 1–2 days.

A closer inspection of the relationship between environmental factors and noise levels (Fig 8) showed that ambient noise levels were lowest during the lowest annual temperatures and highest ice coverage of the austral winter months. We interpret the low noise during this period as due to this increased sea-ice coverage on the open ocean that dampens noise from breaking sea-surface waves. Wind speeds, although high, do not exhibit peak velocities during this time. Noise levels are highest during late summer and fall, when whale vocalization energy is highest, wind speeds exhibit peak values, and ice coverage is increasing but not maximum. To investigate these relations further, we cross-correlated mean noise level with wind speed and temperature in the 51–90 Hz band from the Bransfield Strait data shown in Fig 8. The resulting correlation functions are then shown in Fig 9 Because the peak correlation between noise and wind speed occurs at near zero lag, this implies noise levels are directly correlated with wind speed and therefore noise levels increase as soon as wind speeds increase. Also, since this correlation decreases rapidly as lag increases (both positively and negatively), it suggests the noise effect from wind is immediate and lasts only as long as the wind is sustained. Alternatively, the peak correlation between noise level and air temperature occurs after a 1–2 day lag; however the positive correlation remains high for many days afterward. This suggests that even though the noise impact from temperature is delayed by a few days, it will have a longer-term impact as temperature levels remain at seasonal levels.

### Other Noise Sources

Seafloor earthquakes also contribute some energy to the overall noise spectrum within both the Bransfield Strait [11] and the Scotia Sea [17]. The Bransfield Strait and Scotia Sea have similar volcano-tectonic settings; both are back-arc basins in ocean crust bordered by a chain of island–arc volcanoes above a subduction zone. However, one key difference is that convergence of the adjacent ocean crust is thought to have ceased along the western margin of the Bransfield Strait, and the spreading motion within the back-arc basin has correspondingly slowed [39], whereas the Scotia arc is still a very seismically active, converging subduction zone [40] with 40 earthquakes of magnitude (m_b_) ≥ 5 during the hydrophone deployment period as compared to five similar sized earthquakes in the Bransfield Strait (http://earthquake.usgs.gov/earthquakes/search). The lower pace of plate motion in the Bransfield Strait appears to manifest as a large number of smaller magnitude earthquakes, evidenced by the large number of seismo-acoustic signals recorded on the Bransfield back-arc spreading segments during the hydrophone deployments [11]. However since earthquakes are a temporally random process, it does not seem likely that the different levels of seismic activity between the two regions contribute to the disparity in long-term ambient sound levels.

Although both ships and air gun (for research and/or oil exploration) sounds are occasionally present on the hydrophone data (Fig 3F), they are not evident in the long-term noise spectra, as is seen in the North Atlantic and European Arctic [41]. It is well established that there are much greater levels of ship traffic in the Northern as compared to the Southern Hemisphere [4]. The lower density of ship traffic has been used to explain 20 dB lower average noise levels at some Southern Hemisphere sites [42].

Blue whale sightings are not very common in the Southern Ocean and both blue and fin whale populations are currently at very low levels [43,44] following decades of commercial exploitation during the 20th century [45,46]. The periods of near-continual energy in the blue whale vocalization frequency band we observed over two years in the Bransfield Strait and Drake Passage is consistent with high vocalization rates seen in other studies (e.g. [34]; with 350–2500 detections in just eight days). Blue whale calling peaked in the Drake Passage and Bransfield Strait in late fall when sea ice cover was at a minimum, and decreased through the austral winter as ice cover increased. Moreover, the blue whale call energy correlated with the annual noise levels maxima and minima, suggesting whale vocalizations are a significant, sustained contributor to ambient environmental noise levels. Despite the annual variability in the levels of blue whale vocalization energy, there was a continual year-long presence, as observed in previous studies [8,34]. Interestingly, our interpretation of Fig 8 is that highest overall noise levels in the Bransfield Strait and Drake Passage correlate with maxima in blue whale vocalization energy and wind speeds, but only moderate temperatures and modest ice coverage.

The hydrophone data thus re-affirm the value of acoustic monitoring for establishing the presence of the endangered animal populations [34,47] in areas where extreme weather conditions and ice cover can limit the effectiveness of other (e.g., visual) survey methods. As has been shown elsewhere in the world (e.g., [48]), our study also indicates that baleen whale vocalizations are a major component of the ambient sound field in the Southern Ocean, contributing a similar amount of sound energy as other natural acoustic contributors. Moreover, cetacean vocalizations provide a clear acoustic signal that might be used as a metric to quantify other acoustic stressors introduced into the environment.
